# Latin American Group for Maturity (GDLAM) protocol: a reliable method to assess the functional autonomy of community-dwelling older men

**DOI:** 10.7717/peerj.20412

**Published:** 2026-05-29

**Authors:** Álvaro Huerta Ojeda, Sergio Galdames Maliqueo, Marcela Valderrama Muñoz, Guillermo Barahona-Fuentes, Regina de Villa Garduño, Maximiliano Bravo Yapur, Cristina Gacitúa Ruminot, Mauricio Navarro Vidal, Rolando Salvo Román, Tatiana Pérez Corvalán, María-Mercedes Yeomans-Cabrera, Carlos Jorquera-Aguilera, Emilio Jofré-Saldía

**Affiliations:** 1 Núcleo de Investigación en Salud, Actividad Física y Deporte ISAFYD, Universidad de Las Americas, Viña del Mar, Chile; 2 Laboratorio de Análisis de la Actividad Física y del Deporte, Facultad Ciencias de la Actividad Física y del Deporte, Universidad de Playa Ancha de Ciencias de la Educación, Valparaíso, Chile; 3 Laboratorio de Comportamiento y Desempeño Motor, Universidad de Playa Ancha de Ciencias de la Educación, Valparaíso, Chile; 4 Faculty of Education and Social Sciences, Universidad Andres Bello, Viña del Mar, Chile; 5 Departamento Medicina Interna, Universidad de Valparaíso, Viña del Mar, Chile; 6 Escuela de Psicología, Facultad de Salud y Ciencias Sociales, Universidad de Las Américas, Providencia, Chile; 7 Facultad de Ciencias, Escuela de Nutrición y Dietética, Universidad Mayor, Santiago, Chile; 8 Facultad de Ciencias de la Rehabilitación y Calidad de Vida, Universidad San Sebastián, Santiago, Chile

**Keywords:** Functional autonomy, Latin American group for maturity protocol, Older adults, Older men

## Abstract

**Background:**

Functional autonomy (FA) in older adults can be assessed using the Latin American Group for Maturity Protocol (GDLAM). However, the nter-day reliability of the GDLAM protocol and its General Index of Functional Autonomy (GI) has not been determined in community-dwelling older men (OM).

**Objective:**

To determine the inter-day reliability of the GDLAM protocol and the GI in OM, considering the effect of age on these parameters.

**Materials and methods:**

Fifty OM participated, divided into two age groups (All: 73.9 ± 6.9 years; G1 < 75 years: *n* = 30, 69.2 ± 3.6 years; G2 ≥ 75 years: *n* = 20, 81.0 ± 3.9 years). A repeated measures design was used to analyze the inter-day reliability of the five functional tests of the GDLAM protocol (in seconds) and the GI (in points). The statistical analysis included the intraclass correlation coefficient (ICC) and the coefficient of variation (CV). Reliability was considered acceptable when CV ≤ 10% and ICC ≥ 0.80, and high when CV ≤ 5% and ICC ≥ 0.90.

**Results:**

The overall analysis and analysis by age group showed high inter-day reliability (All: CV = 4.38%, ICC = 0.91; G1: CV = 3.35%, ICC = 0.93; G2: CV = 4.33%, ICC = 0.92).

**Conclusion:**

The GDLAM protocol and GI show high inter-day reliability and reproducibility, supporting their use to assess FA in community-dwelling older adults.

## Introduction

The human aging process involves changes in various bodily systems (Angulo et al., 2020). In this context, because protein metabolism changes from aging, one of the most affected systems is the musculoskeletal system (Sartori, Romanello & Sandri, 2021). In this regard, it has been demonstrated that both exercise capacity and resistance to physical stress decline with age (Moreno González, 2005). Indeed, scientific evidence has shown that a loss of musculoskeletal function caused by aging is associated with an increased risk of developing frailty syndrome (Angulo et al., 2020), a clinical state characterized by a decrease in functional reserve and the ability to adapt to stress, which in turn increases the risk of adverse health outcomes. This condition is caused by the simultaneous alteration of several physiological processes, including oxidative stress and inflammation, which affect numerous organs and systems, such as the respiratory and cardiovascular systems (Angulo et al., 2020). In practice, frailty can significantly impact the ability to perform basic and functional activities of daily living, resulting in a reduction in functional autonomy, independence, and quality of life for those affected (Dent et al., 2019). Therefore, it is imperative to assess and monitor the functional autonomy of older adults who are functionally independent or in the early stages of frailty syndrome, in a valid and reliable manner (Park & Ko, 2021).

The aging phenomenon affects both men and women. However, when comparing the two sexes, some differences in markers of autonomy have been identified (Calatayud et al., 2021; Deore et al., 2023). In this context, it has been shown that older men have greater muscle mass and strength than women (Huang et al., 2025). Similarly, older men perform better than women on tests that assess and evaluate activities of daily living (Calatayud et al., 2021). However, evidence also shows that men are frailer than women, and that comorbidities such as hypertension and coronary heart disease contribute to this frailty, with cognitive decline and reduced autonomy (Deore et al., 2023). Therefore, regardless of whether older men have better functional indicators than older women, they should undergo functional autonomy assessments before entering a state of frailty or dependency.

Within this framework, functional autonomy is recognized as an indicator of physical health in older adults (Dantas & Vale, 2004). This concept refers to the ability to carry out daily activities without external assistance, which is essential for preserving both independence and quality of life (Faustino, Gandolfi & De Azevedo Moura, 2014). For this reason, it is vital to maintain this capacity over time, particularly in the advanced stages of life. In terms of prevention, it is essential to assess this indicator in older people continually (Barry et al., 2014). In this context, the early detection of any signs of deterioration in functional autonomy enables the implementation of individualized and effective intervention strategies to maintain functionality, delay the onset of dependency, and improve quality of life (Muñoz, Rojas & Marzuca, 2015).

Over time, and depending on the application context, various tests and protocols have been used to assess the physical health of older people (Barry et al., 2014; De Fátima Ribeiro Silva et al., 2021; Huerta Ojeda et al., 2024a). Among these, to determine the functional autonomy of older people, the Latin American Maturity Group (GDLAM) protocol has been used, a battery specifically designed to assess functional autonomy in this age group (Dantas et al., 2014; Vale, 2005). This protocol involves the application of five functional tests that represent the activities performed by an independent person in daily life. The five tests are timed and measured in seconds. After the five tests have been performed, a formula is used to calculate the General Functional Autonomy Index, where a lower score corresponds to greater functional autonomy (Vale, 2005). In practical terms, the GDLAM protocol has proven to be more sensitive than other functional tests, such as the Short Physical Performance Battery (SPPB), especially in older people with higher levels of physical performance (Bergland & Strand, 2019). Furthermore, as it is a battery of tests aimed at community-dwelling older people, the GDLAM protocol can be correlated with functional impairments such as limb asymmetries (Huerta Ojeda et al., 2022) or serve as an objective marker of musculoskeletal deterioration associated with aging (Jofré-Saldía et al., 2024). Originally, the GDLAM protocol was developed in Brazil (Vale, 2005) and has been used in isolation in various countries, including North America (Ochoa Martínez et al., 2015), Spain (Marcos-Pardo et al., 2020), Chile (Huerta Ojeda et al., 2024a; Huerta Ojeda et al., 2024b), and Brazil itself (Vale, 2005). In the same context, a test-retest reliability study of the GDLAM protocol in community-dwelling older women was published in 2024 (Huerta Ojeda et al., 2024a). Based on this background, the usefulness of GDLAM has been demonstrated for monitoring functional autonomy in both public and private intervention programs, allowing for consistent evaluations over time and accurate estimates of the impact of such programs (Galdames Maliqueo et al., 2025). However, to date, no study has been conducted to determine the reliability of the GDLAM protocol in older men, which limits its use as a diagnostic tool in this population. Therefore, the main objective of this study was to determine the inter-day reliability of the GDLAM protocol and the General Index of Functional Autonomy (GI) in community-dwelling older men. As a secondary objective, the effect of aging on this reliability was analyzed. The hypothesis was that both the GDLAM protocol and the GI have high inter-day reliability, regardless of the age group in which they are applied.

## Materials and Methods

### Research design

**Figure 1:**
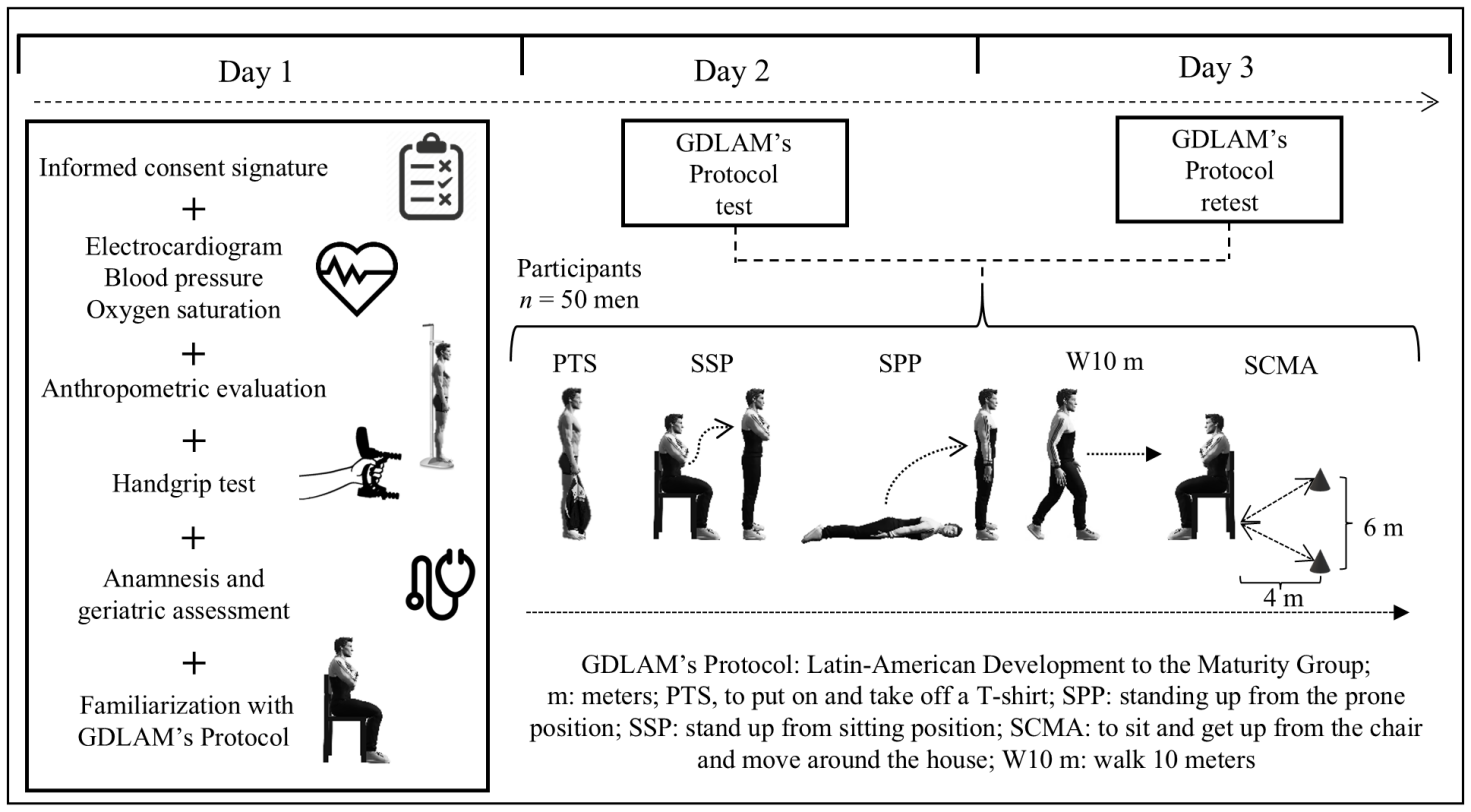
Research design. GDLAM’s Protocol, Latin-American Development to the Maturity Group; m, meters; PTS, to put on and take off a T-shirt; SPP, standing up from the prone position; SSP, stand up from sitting position; SCMA, to sit and get up from the chair and move around the house; W10 m, walk 10 m.

This cross-sectional study employed a repeated-measures design to evaluate inter-day test–retest reliability, incorporating the five tests of the GDLAM protocol and the GI. The order of the GDLAM protocol tests was standardized (see GDLAM’s Protocol section). For all participants, measurements were taken over three days, separated by 48 h (Fig. 1). During this period, participants were asked to refrain from exercise or any other activity that would cause fatigue. Once the data had been recorded, the participants were divided into two groups to determine the possible effects of aging on reliability thresholds: Group 1, comprising participants aged 60.0–74.9 years, and Group 2, comprising participants aged 75.0 years or older.

### Participants

To estimate the sample size, the statistical program G*Power (v3.1.9.7, Heinrich-Heine-Universität, Germany) was used (Faul, 2020). The methodology combined three tests: (a) *t*-test, (b) Means: Difference between two dependent means (matched pairs), and (c) *A priori*: Compute required size –given *α*, power, and effect size. Next, the following parameters were introduced: two tails, effect size *dz* = 0.41, *α*-error < 0.05, and a power (1-*β* error) = 0.8. This calculation indicated that the sample size required for the study was equivalent to 49 participants.

Fifty older men, aged between 62 and 90, voluntarily participated in this study. Subsequently, to fulfill the secondary objective of the research, the sample was divided into Group 1 (*n* = 30, 69.2 ± 3.6 years) and Group 2 (*n* = 20, 81.0 ± 3.9 years). Based on the history obtained during study enrollment, participants in both groups were classified as sedentary, as they did not meet the minimum activity guidelines or their physical activity was occasional and/or incidental (*e.g.*, walking to work, household activities) (McKay et al., 2022). The inclusion criteria were: being male, over 60 years of age, able to move independently, and capable of giving consent. Those who were terminally ill, had pre-existing cardiovascular conditions or conditions detected by electrocardiogram, severe lung conditions, recent fractures (within the last three months), neurodegenerative diseases, severe dementia, physical limitations that prevented them from performing specific tests, or who refused to sign the informed consent form were excluded from the study. However, no one was excluded. All participants read and signed the informed consent form. The recruitment and evaluation of participants took place between April and May 2025. Informed consent was provided in print and signed before the assessments began. All participants were informed of the study’s purpose. Both the research protocol and the consent form were validated by the Ethics and Scientific Committee of the University of Las Américas, Chile (registration number CEC_PI_2023003). The study followed the ethical guidelines for sports and exercise sciences (Harriss, MacSween & Atkinson, 2019)*.*

### Anthropometric measurements and handgrip test

To characterize the sample, body mass, height, body mass index (BMI), body fat percentage, and handgrip strength were measured using an impedance measurement (Tanita Inner Scan, Model BC-554^®^; Tanita, Tokyo, Japan). Maximal hand strength was assessed using a hand dynamometer (Jamar Plus + Hand dynamometer model, Performance Health; Downers Grove, IL, USA) (Mathiowetz, 2002). Before starting, the dynamometer was adjusted to the size of each participant’s hand (Ruiz-Ruiz et al., 2002). The test was performed with participants seated, with their arms at their sides, elbows bent at 90°, and forearms in a neutral position. The isometric contraction of maximum grip strength was maintained for 3 to 5 s (Arroyo et al., 2007; Iconaru et al., 2018). Participants made two attempts with each hand (dominant and non-dominant), with the best score from each hand used for statistical analysis.

### GDLAM’s protocol

The functional autonomy of participants was assessed using the GDLAM protocol (Andrade et al., 2015; Dantas et al., 2014; Dantas & Vale, 2004; Vale, 2005), which includes five functional tests: (1) To put on and take off a T-shirt (PTS), (2) Stand up from sitting position (SSP), (3) Standing up from the prone position (SPP), (4) walk 10 m (W10 m), and (5) To sit and get up from the chair and move around the house (SCMA). To ensure the study was conducted correctly, participants attended a session to familiarize themselves with the GDLAM protocol, which includes five functional tests. Then, in a second session, the measurement (test) was taken, followed by a third session (retest) 48 h later. In both the test and retest, participants were given only one attempt per exercise and were instructed to perform as quickly as possible without compromising their physical safety. The rest period between exercises was 3 min. The GDLAM protocol was applied by experienced evaluators, with more than 400 evaluations of older women associated with another research project on functional autonomy. All tests were measured in seconds (s), while GI was quantified in points using the following equation: GI = [(PTS + SSP + SPP + W10 m) × 2 + SCMA]/4. A digital stopwatch (model HS-70W-1; Casio^®^, Tokyo, Japan) was used to record the time. The description of the five functional tests was carried out by Huerta Ojeda et al. (2024a) and Huerta Ojeda et al. (2024b) and registered at https://osf.io/dby4s/.

### Data analysis

Descriptive information on the five functional tests of the GDLAM protocol, GI, handgrip strength, and anthropometric variables, is presented as means and standard deviations (SD). Data normality was verified using the Shapiro–Wilk test (*p* > 0.05). For parametric data, comparisons of age, anthropometric measurements, and grip strength between groups were performed using an independent samples *t*-test; for non-parametric data, the Mann–Whitney U test was used. For all 50 participants and Groups 1 and 2, absolute reliability was estimated using the standard error of measurement (SEM) and the coefficient of variation (CV), while relative reliability was assessed using the intraclass correlation coefficient (ICC), model 3.1 (Weir, 2005). All these estimates were made in a customized spreadsheet (Hopkins, 2015)*.* An ICC ≥ 0.80 was considered acceptable, and an ICC ≥ 0.90 was considered high; for CV, thresholds of ≤ 10% were established for acceptance, and CV ≤ 5% for high reliability (James et al., 2017). To examine the relationship between the GDLAM and GI test and retest, Pearson’s correlation coefficient was applied (Hopkins et al., 2009), interpreting the values of *r* with the following ranges: trivial (<0.1), small (0.1−0.3), moderate (0.3−0.5), high (0.5−0.7), very high (0.7−0.9), or practically perfect (>0.9) (Hopkins et al., 2009). However, as this last test does not allow systematic errors to be detected (Weir, 2005), the Bland–Altman method was used to identify possible systematic biases (Bland & Altman, 1986), recommended for use as a complement to Pearson’s correlation (Weir, 2005). All analyses were performed using Prism 10.5.0 software (Laguna Hills, CA, USA) for Windows (Microsoft, Redmond, WA, USA), with a 95% confidence level and a statistical significance of *p* < 0.05.

## Results

At the time of the study, the 50 participants had an average age of 73.9 ± 6.9 years. Anthropometric analysis revealed a body mass of 78.9 ± 13.2 kg, a height of 166.8 ± 6.0 cm, and a BMI of 28.3 ± 4.1 kg/m^2^. When comparing the anthropometric characteristics between Group 1 and Group 2, both age and grip strength showed significant differences (*p* < 0.05). Body mass, height, BMI, and fat mass percentage showed no statistically significant differences between the two groups (*p* > 0.05). The anthropometric characteristics of the 50 participants, as well as the comparison between Group 1 and Group 2, are detailed in Table 1.

**Table 1:** Sample characteristics, mean values, and differences between Group 1 and Group 2.

Variables	All *n* = 50		Group 1 *n* = 30		Group 2 *n* = 20		Comparison between groups				
	mean ± SD min–max		mean ± SD min–max		mean ± SD min–max		*t*	*p*	Diff ± SEM	95% CI	
Age^**^ years	73.9 ± 6.9 62.0–90.0		69.2 ± 3.6 62.0 –75.0		81.0 ± 3.9 76.0–90.0		10.77	<0.001	11.73 ± 1.09	9.54 to 13.92	
Body mass^*^ kilograms	78.9 ± 13.2 50.1–125.6		81.4 ± 15.5 50.1–125.6		75.1 ± 7.8 58.8–87.2		1.68	ns	−6.33 ± 3.76	−13.91 to 1.23	
Stature^*^ centimeters	166.8 ± 6.0 155.0–183.0		166.8 ± 6.2 155.0–183.0		166.8 ± 5.8 159.0–180.0		<0.01	ns	0.01 ± 1.75	−3.51 to 3.53	
BMI^**^ kg/m^2^	28.3 ± 4.1 18.0–42.5		29.2 ± 4.8 18.0–42.5		27.0 ± 2.4 20.8–31.2		1.88	ns	−2.20 ± 1.17	−4.56 to 0.15	
Fat mass^*^ percentage	37.3 ± 7.7 13.1–53.7		38.3 ± 7.8 13.1–50.5		35.8 ± 7.3 22.2–53.7		1.12	ns	−2.49 ± 2.21	−6.95 to 1.96	
Strength D^*^ N	337.4 ± 57.5 215.8–456.2		352.3 ± 50.8 240.3–444.4		315.0 ± 60.8 215.8–456.2		2.35	0.022	−37.35 ± 15.88	−69.29 to −5.41	
Strength Non-D^*^ N	321.3 ± 65.1 147.2–464.0		337.3 ± 56.5 239.4–449.3		297.4 ± 71.2 147.2–464.0		2.20	0.032	−39.92 ± 18.12	−76.35 to −3.48	

**Notes.**

BMI body mass index D dominant hand Diff difference between means Group 1 participants under 75 years of age Group 2 participants 75 years of age or older max maximum Non-D non-dominant hand min minimum ns no significative SEM standard error of the medians SD standard deviation

* The comparison between groups, the *t*-test was used.

** The comparison between groups, the Mann–Whitney test was used.

In line with the main objective of the study, the first analysis showed that the average values of the 50 participants in the five functional tests and the GI were within the “Fair” and “Good” categories, with only the SPP test falling within the “Poor” category (Araújo Gomez, 2024). Subsequently, the analysis revealed that two tests of the GDLAM protocol exceeded the acceptable threshold for CV: PTS = 11.47% and SPP = 11.80%. Meanwhile, the SSP test was within the acceptable threshold between the test and retest (SSP = 8.39%). Finally, the W10 m and SCMA tests showed a CV equivalent to 4.93 and 4.70%, respectively, classifying them as having a high CV. Regarding the ICC, all five tests showed an acceptable threshold (≥ 0.80). When evaluating the GI, a CV of 4.38% was obtained, considered within a high range, and an ICC of 0.91, also classified as high. These results suggest that, overall, the GDLAM protocol has low systematic error and high interday reproducibility. The mean values, standard deviations, and 95% confidence intervals (95% CI) are detailed in Table 2.

**Table 2:** Test–retest reliability of the GDLAM protocol (*n* = 50).

Variables	Test (mean ± SD)	Retest (mean ± SD)	*p*	Δ[95% CI]	SEM [95% CI]	CV [95% CI]	ICC [95% CI]
PTS (s)	13.89 ± 3.84	12.57 ± 3.01	<0.0001	−1.31 [−1.92 to −0.70]	1.51 [1.26–1.89]	11.47 [9.58–14.29]	0.81 [0.69–0.88]
SSP (s)	9.93 ± 2.07	9.59 ± 2.00	0.0479	−0.33 [−0.66 to 0.00]	0.82 [0.68–1.02]	8.39 [7.01–10.46]	0.84 [0.73–0.90]
SPP (s)	3.62 ± 1.08	3.44 ± 0.91	0.0347	−0.18 [−0.34 to −0.01]	0.41 [0.34–0.52]	11.80 [9.86–14.70]	0.83 [0.72–0.90]
W10 m (s)	6.09 ± 0.70	5.70 ± 0.75	<0.0001	−0.38 [−0.50 to −0.26]	0.29 [0.24–0.36]	4.93 [4.12–6.15]	0.84 [0.74–0.90]
SCMA (s)	41.37 ± 5.80	38.65 ± 4.78	<0.0001	−2.72 [−3.48 to −1.97]	1.88 [1.57–2.34]	4.70 [3.93–5.86]	0.87 [0.79–0.92]
GI (points)	27.11 ± 4.17	25.32 ± 3.56	<0.0001	−1.79 [−2.25 to −1.32]	1.14 [0.95–1.43]	4.38 [3.66–5.46]	0.91 [0.85–0.95]

**Notes.**

CI confidence interval ICC intra-class correlation coefficient CV coefficient of variation GDLAM’s protocol Latin-American Development to the Maturity Group GI GDLAM index of autonomy *p* *p*-value PTS to put on and take off a T-shirt s seconds SCMA to sit and get up from the chair and move around the house SD standard deviation SEM standard error of the mean SPP standing up from the prone position SSP standing up from sitting position W10 m walking 10 m Δ variation delta

**Figure 2:**
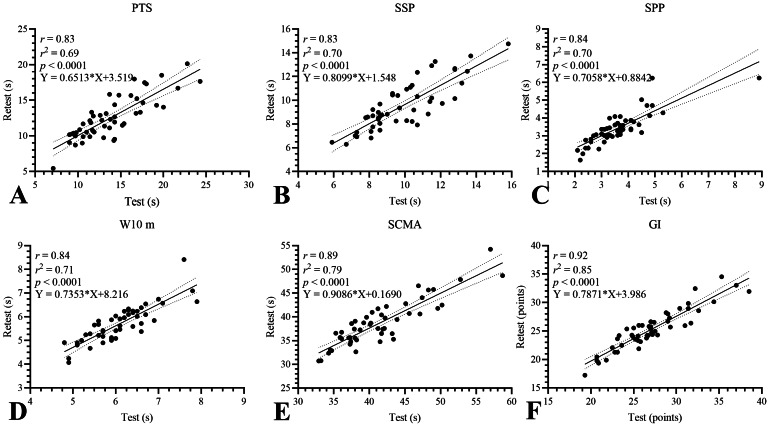
Regression analysis for GDLAM’s protocol between test-retest. GDLAM’s protocol, Latin-American Development to the Maturity Group; GI, GDLAM index of autonomy; PTS, to put on and take off a T-shirt; s, seconds; SCMA, to sit and get up from the chair and move around the house; SPP, standing up from the prone position; SSP, standing up from sitting position; W10 m, walking 10 m.

Pearson’s correlation analysis between test-retest indicates that the five tests in the GDLAM protocol achieved a very high level of correlation (0.7−0.9): PTS *r* = 0.83 (95% CI [0.71–0.90]), SSP *r* = 0.83 (95% CI [0.73–0.90]), SPP *r* = 0.84 (95% CI [0.73–0.90]), W10 m *r* = 0.84 (95% CI [0.73–0.90]) and SCMA *r* = 0.89 (95% CI [0.81–0.93]). When evaluating GI, an *r* = 0.92 (95% CI [0.86–0.95]) was observed, indicating that the GDLAM protocol, as a whole, exhibits high inter-day agreement. Figure 2 graphically presents the results of the Pearson test, including the regression line, the r^2^ value, the level of significance, and the corresponding equation.

**Figure 3:**
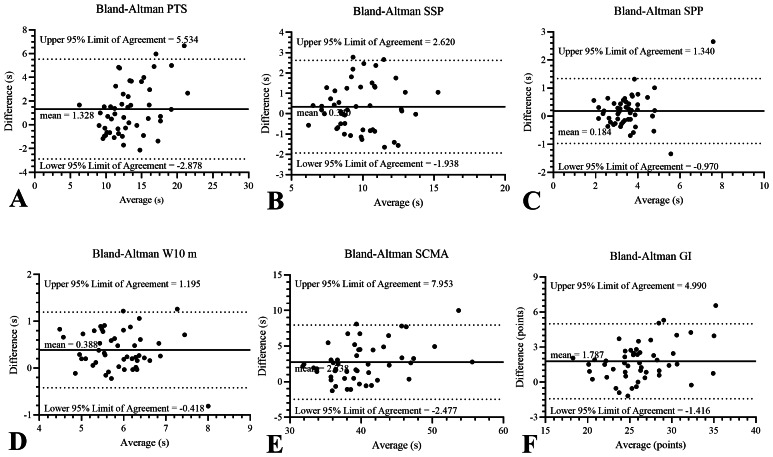
Bland-Altman analysis. The solid line represents the average of the differences between variables evaluated through the GDLAM’s protocol in test-retest (*n* = 50). The segmented lines represent 95% of the upper and lower confidence limits. GDLAM’s protocol, Latin-American Development to the Maturity Group; GI, GDLAM index of autonomy; PTS, to put on and take off a T-shirt; s, seconds; SCMA, to sit and get up from the chair and move around the house; SPP, standing up from the prone position; SSP, standing up from sitting position; W10 m, walking 10 m.

When comparing the mean values and differences between the test-retest of the GDLAM protocol and the GI, the Bland-Altman analysis revealed the following common biases: PTS = 1.32 ± 2.14 s, SSP = 0.34 ± 1.16 s, SPP = 0.18 ± 0.58 s, W10 *m* = 0.38 ± 0.41 s, SCMA = 2.73 ± 2.66 s, and GI = 1.78  ± 1.63 points. It is worth noting that the observed biases are small in comparison to the average times in each of the GDLAM and GI protocol tests. Figure 3 illustrates the graphical representation of this analysis, which includes the mean difference line and the upper and lower lines corresponding to the 95% confidence interval.

In line with the secondary objective of the study, the average values of participants belonging to Group 1 showed a GI classified as “Regular” and “Good” for the test and retest, respectively; while participants belonging to Group 2 showed a GI classified as “Good” and “Very Good” for the test and retest, respectively (Araújo Gomez, 2024). Then, when analyzing the five GDLAM protocol tests performed by Group 1, it was observed that three of them were within the acceptable threshold for CV (PTS = 9.79%, SSP = 8.25%, and SPP = 9.72%). Meanwhile, the W10 m and SCMA tests were within the high threshold between the test and retest (W10 *m* = 4.33% and SCMA = 3.44%). Regarding the ICC, all five tests demonstrated an acceptable threshold (≥ 0.80). When evaluating the GI of Group 1, a CV of 3.35% and an ICC of 0.93 were observed, both of which are classified as high. Similarly, when analyzing the five GDLAM protocol tests performed by Group 2, it was observed that two of them were outside the acceptable threshold for CV (PTS = 11.77% and SPP = 13.26%). The SSP, W10m, and SCMA tests were within the acceptable threshold between the test and retest, with a coefficient of variation ≤ 10.0%. Regarding the ICC, it was found that all five tests had an acceptable threshold (≥ 0.80). When evaluating the GI of Group 2, a CV equivalent to 4.33% and an ICC of 0.92 were observed, both of which are classified as high. These results suggest that, regardless of the age range of men in which the GDLAM protocol is applied, it has low systematic error and high inter-day reproducibility. The mean values, standard deviations, and 95% confidence intervals for Group 1 and Group 2 are detailed in Table 3.

**Table 3:** Test–retest reliability of the GDLAM protocol for Group 1 and Group 2.

Variables	Test (mean ± SD)	Retest (mean ± SD)	*p*	Δ[95% CI]	SEM [95% CI]	CV [95% CI]	ICC [95% CI]
Group 1							
PTS (s)	12.84 ± 3.47	12.16 ± 2.78	0.0409	−0.67 [−1.32 to −0.02]	1.22 [0.97–1.64]	9.79 [7.79–13.16]	0.85 [0.72–0.92]
SSP (s)	9.70 ± 2.00	9.55 ± 1.94	0.4563	−0.15 [−0.57 to 0.26]	0.79 [0.63–1.06]	8.25 [6.57–11.09]	0.84 [0.70–0.92]
SPP (s)	3.36 ± 0.77	3.31 ± 0.95	0.4924	−0.05 [−0.22 to 0.11]	0.32 [0.25–0.43]	9.72 [7.74–13.08]	0.86 [0.74–0.93]
W10 m (s)	5.97 ± 0.57	5.53 ± 0.66	<0.0001	−0.43 [−0.56 to −0.30]	0.24 [0.19–0.33]	4.33 [3.45–5.82]	0.84 [0.70–0.92]
SCMA (s)	39.36 ± 3.89	37.5 ± 3.86	<0.0001	−1.85 [−2.55 to −1.16]	1.32 [1.05–1.77]	3.44 [2.74–4.62]	0.89 [0.78–0.94]
GI (points)	25.78 ± 3.39	24.65 ± 3.24	<0.0001	−1.12 [−1.57 to −0.68]	0.84 [0.67–1.13]	3.35 [2.67–4.51]	0.93 [0.87–0.97]
Group 2							
PTS (s)	15.46 ± 3.91	13.18 ± 3.30	<0.0001	−2.27 [−3.39 to −1.16]	1.68 [1.28–2.46]	11.77 [8.95–17.19]	0.80 [0.56–0.91]
SSP (s)	10.26 ± 2.17	9.66 ± 2.13	0.0361	−0.59 [−1.15 to −0.04]	0.84 [0.63–1.22]	8.44 [6.42–12.33]	0.86 [0.68–0.94]
SPP (s)	4.01 ± 1.36	3.65 ± 0.82	0.0346	−0.36 [−0.7 to −0.02]	0.5 [0.38–0.74]	13.26 [10.08–19.37]	0.81 [0.58–0.92]
W10 m (s)	6.27 ± 0.84	5.96 ± 0.83	0.0112	−0.30 [−0.53 to −0.07]	0.34 [0.26–0.50]	5.62 [4.27–8.21]	0.84 [0.65–0.93]
SCMA (s)	44.40 ± 6.90	40.36 ± 5.58	<0.0001	−4.03 [−5.51 to −2.55]	2.23 [1.69–3.26]	5.26 [4.00–7.69]	0.88 [0.73–0.95]
GI (points)	29.11 ± 4.52	26.32 ± 3.85	<0.0001	−2.78 [−3.57 to −1.98]	1.20 [0.91–1.75]	4.33 [3.29–6.33]	0.92 [0.82–0.97]

**Notes.**

CI confidence interval ICC intra-class correlation coefficient CV coefficient of variation GDLAM’s protocol Latin-American Development to the Maturity Group Group 1 60.0–74.9 years range (*n* = 30) Group 2 ≥ 75.0 years range (*n* = 20) GI GDLAM index of autonomy *p* *p*-value PTS to put on and take off a T-shirt s seconds SCMA to sit and get up from the chair and move around the house SD standard deviation SEM standard error of the mean SPP standing up from the prone position SSP standing up from sitting position W10 m walking 10 m Δ variation delta

## Discussion

This study determined the inter-day reliability of the GDLAM protocol and the GI in community-dwelling older men. The findings revealed that both the GDLAM protocol and the GI have high inter-day agreement and reproducibility. The results also showed that the inter-day agreement and reproducibility of the GDLAM protocol and the GI are not affected by age. Therefore, based on the results, it can be observed that the GDLAM protocol and GI are highly reliable for measuring functional autonomy in older men, regardless of the age range evaluated, thus accepting the proposed hypothesis.

### To put on and take off a T-shirt

The PTS test assesses the ability to dress and undress, also observing shoulder girdle mobility and upper limb coordination (Vale, 2005). In the GDLAM protocol, this test is measured in seconds, with no alternative scoring methods (Vale et al., 2006). In its initial validation, it showed medium-high reliability (*r* = 0.759, *p* < 0.01) (Vale et al., 2006). In this regard, the only test-retest reliability study of the GDLAM protocol focused on community-dwelling older women, showing a CV = 11.22 and an ICC = 0.86 (Huerta Ojeda et al., 2024a). In the present study, a CV of 11.47% (95% CI [9.58–14.29]) and ICC = 0.81 (95% CI [0.69–0.88]) were observed. The group analysis revealed that both Group 1 and Group 2 obtained values like those of the overall analysis (G1: CV = 9.79, ICC = 0.85; G2: CV = 11.77, ICC = 0.80). Furthermore, given that this test assesses a daily living activity essential for personal independence, related to joint mobility, and upper limb coordination, it is considered a key component of the GDLAM protocol (Vale, 2005).

### Stand up from the sitting position

The SSP test assesses lower limb strength, which is crucial in determining a person’s physical abilities, particularly in demanding, high-energy activities such as transitioning from a sitting to a standing position (Hatamoto et al., 2016; Jerez-Mayorga et al., 2021; Riley et al., 1991). Additionally, this test has been linked to greater functional autonomy, reduced fear of falling, and decreased difficulty climbing stairs (Huerta Ojeda et al., 2022; Löppönen et al., 2022). In the GDLAM protocol, five repetitions are evaluated (Vale, 2005). However, it can also be applied independently, quantifying both the force (kg or N) and the speed of execution in its propulsive phase (cm s^−^^1^) (Jerez-Mayorga et al., 2021). In a study conducted by Guralnik et al. (1994), this test was initially validated in older adults, highlighting its usefulness in determining functional autonomy in this age group. A recent study focusing on community-dwelling older women in the SSP reported a CV = 9.01 and an ICC = 0.79, demonstrating acceptable reliability and high inter-day reproducibility (Huerta Ojeda et al., 2024a). In the present study, the overall analysis of the SSP test (*n* = 50) showed a CV = 8.39% (95% CI [7.01–10.46]) and ICC = 0.84 (95% CI [0.73–0.90]), with similar values in groups 1 and 2 (see Table 3), indicating acceptable reliability and high inter-day reproducibility in community-dwelling older men, regardless of the age range evaluated. Despite the encouraging results and the evidence presented by this test in isolation, the SSP alone is not sufficient to determine the functional autonomy of older men. Therefore, integrating other functional tests, such as those in the GDLAM protocol, would allow for a more accurate determination of the functional autonomy of this age group (Vale, 2005).

### Stand up from the prone position (SPP)

The SPP test evaluates the ability to get up from the floor in a prone position without assistance, assessing physical skills such as strength, flexibility, coordination, and balance (Alomar et al., 2020; Vale, 2005). This test can be evaluated in seconds, as in the GDLAM protocol (Vale, 2005) or through scores (Alomar et al., 2020). Its difficulty is particularly significant for older people living in nursing homes, where it is a limiting factor in maintaining functional autonomy (Alexander et al., 1997; Vale, 2005). In addition, SPP has been associated with lower mortality: +1 point implies a 21% lower risk (Barreto de Brito et al., 2014). Specifically, a reliability study conducted in women revealed that the SPP achieved a CV = 9.01 and an ICC = 0.79, demonstrating acceptable reliability and high inter-day reproducibility (Huerta Ojeda et al., 2024a). The reliability analysis of the present study showed that the SPP test has a CV = 11.80% (95% CI [9.86–14.70]) and ICC = 0.83 (95% CI [0.72–0.90]), with similar values for both G1 and G2. The SPP is a safe and straightforward tool for assessing the functional autonomy of community-dwelling individuals (Barreto de Brito et al., 2014; Vale, 2005), although it is not sufficient on its own to fully determine functional autonomy.

### Walk 10 m

The W10 m test evaluates linear displacement and, implicitly, dynamic balance and the risk of falls (Barry et al., 2014; Sipilä et al., 1996), simulating walking, which is considered one of the most representative daily activities of a person’s independence. It can be applied within the GDLAM protocol (in seconds) (Vale, 2005) or independently (in m s^1^) (Chan & Pin, 2019). Historically, it has shown high inter-day reproducibility (CV < 5%) (Sipilä et al., 1996), with high levels of reliability (ICC > 0.98) (Chan & Pin, 2019; Peters, Fritz & Krotish, 2013). Along the same lines, the test-retest reliability study of the GDLAM protocol in community-dwelling older women showed a CV = 8.30 and an ICC = 0.74 (Huerta Ojeda et al., 2024a). In the present study, the W10 m test obtained a CV of 4.93% (95% CI [4.12–6.15]) and an ICC = 0.84 (95% CI [0.74–0.90]). Meanwhile, the group analysis revealed that both Group 1 and Group 2 obtained values comparable to those of the overall analysis. (G1: CV = 4.33, ICC = 0.84; G2: CV = 5.62, ICC = 0.84). These results indicate acceptable reliability for W10 m. However, applying this test in isolation is insufficient to determine the functional autonomy of older men; therefore, additional tests are required (Vale, 2005).

### Sit, get up from the chair, and move around the house

The SCMA test assesses agility and balance in everyday activities, such as getting into a car or standing up quickly. Its development on multiple planes and axes, together with the variation of the center of mass, makes it representative of everyday life (Andreotti & Okuma, 1999). The SCMA test is measured in seconds (Vale, 2005) and, to the best of our knowledge, it has no known alternative forms of punctuation (Andreotti & Okuma, 1999). During the test, walking speed, balance, agility, and implicit muscle strength are observed. Due to these characteristics, the SCMA test is essential in the GDLAM protocol, as it allows for the simulation of movements within the home and functional activities typical of daily life (Huerta Ojeda et al., 2024b). This test was validated by Andreotti & Okuma (1999), demonstrating high reliability (ICC = 0.99; *r* = 0.96). Subsequently, in a reliability study in community-dwelling older women, the SCMA test showed a CV = 4.95 and an ICC = 0.90 (Huerta Ojeda et al., 2024a). In the present study, the CV of the SCMA test was 4.70% (95% CI [3.93–5.86]) and the ICC was 0.87 (95% CI [0.79–0.92]). In addition, the group analysis yielded reliability values comparable to the overall values (G1: CV = 3.44, ICC = 0.89; G2: CV = 5.26, ICC = 0.88).

### GDLAM index of autonomy

The GI is a numerical indicator that reflects the level of functional autonomy of the people assessed using the five tests of the GDLAM protocol. It is calculated using an equation that integrates the results of these tests, where lower values correspond to higher levels of functional autonomy and identify people with greater independence in carrying out daily activities (Vale, 2005). One of the main strengths of both the tests included in the GDLAM protocol and the GI is the absence of a ceiling effect (Bergland & Strand, 2019), since all tests are expressed in seconds, allowing performance improvements to be recorded regardless of the age or gender of the participants.

In addition to the above, a comparative advantage of the GDLAM protocol over other field tests aimed at older adults is that it was created for community-dwelling individuals rather than in institutions (Barry et al., 2014). Subsequently, based on this index, qualitative scales have been developed to classify the functional autonomy of older people in different countries (Dantas et al., 2014; Marcos-Pardo et al., 2020). Specifically, the reliability of this index was calculated for the first time in a study targeting community-dwelling older women, showing a CV = 6.00 and an ICC = 0.91 (Huerta Ojeda et al., 2024a). Similarly, the reliability results of the present study showed a CV of 4.38% (95% CI [3.66–5.46]) and an ICC = 0.91 (95% CI [0.85–0.95]), with no observable variations between groups of different age ranges (G1: CV = 3.35, ICC = 0.93; G2: CV = 4.33, ICC = 0.92).

These results indicate acceptable reliability and high inter-day reproducibility for the GI. Therefore, the GDLAM protocol and the GI adequately meet reliability thresholds for accurately assessing the functional autonomy of community-dwelling older men, regardless of the age range assessed. Finally, the GI not only quantitatively summarizes functional performance but also provides a valuable tool for longitudinal monitoring of functional autonomy, facilitating clinical and intervention decisions without the need to interpret each test separately.

### Limitations

This study included men aged 60 years and older. Therefore, the results apply to that segment of the older population. However, some initiatives have evaluated functional autonomy using the GDLAM protocol in younger populations (50.0–59.9 years). In this context, the reliability thresholds of the GDLAM protocol in younger groups are unknown. Furthermore, as the present study was conducted in community-dwelling older men, the performance of this protocol in institutionalized older adults is unknown. Also, although adequate levels of reliability were observed, sensitivity to change and concurrent validity with other functional instruments or tests were not evaluated. Additionally, to determine its convergent validity, it is recommended to compare the GI with other standardized instruments that assess functional autonomy. Finally, the significant differences observed between the test and retest in three of the five tests from the GDLAM and GI protocols may be attributed to a motor learning process by the participants. Therefore, future research should consider including additional familiarization sessions prior to the administration of the initial tests.

## Conclusions

The results of this study demonstrate that the five tests of the GDLAM protocol and the GI exhibit high inter-day reliability and reproducibility, thereby supporting the acceptance of the research hypothesis. Therefore, the GDLAM protocol is reliable for assessing the functional autonomy of community-dwelling older men.

### Perspective

A future line of work could focus on determining the inter-day reliability of the GDLAM protocol in individuals aged ≤ 60 years. In this way, and considering that the symptoms of aging manifest themselves before reaching older adulthood, the GDLAM protocol could be applied as part of the diagnosis and intervention of younger people, providing a reliable indicator for functional autonomy assessment processes. On the other hand, a second line of work could aim to validate this instrument in institutionalized older adults, thereby expanding the protocol’s coverage. The GDLAM protocol could also be incorporated into community health programs or annual geriatric checkups as a tool for monitoring functional autonomy and timely referral to preventive or rehabilitative interventions in older men. Finally, future research could investigate the sensitivity to change and concurrent validity of the five tests from the GDLAM protocol in comparison to other instruments and functional tests.

##  Supplemental Information

10.7717/peerj.20412/supp-1 Supplemental Information 1 STROBE checklist

## Additional Information and Declarations

### Data Availability

The following information was supplied regarding data availability: The data is available at Figshare: Huerta, Álvaro (2025). Dataset - Reliability GDLAM protocol (men). figshare. Dataset. https://doi.org/10.6084/m9.figshare.29647499.v1.
